# Pharmmaker: Pharmacophore modeling and hit identification based on druggability simulations

**DOI:** 10.1002/pro.3732

**Published:** 2019-12-04

**Authors:** Ji Young Lee, James M. Krieger, Hongchun Li, Ivet Bahar

**Affiliations:** ^1^ Department of Computational and Systems Biology School of Medicine, University of Pittsburgh Pittsburgh Pennsylvania

**Keywords:** druggability, molecular dynamics, pharmacophore modeling, target‐based drug discovery, virtual screening

## Abstract

Recent years have seen progress in druggability simulations, that is, molecular dynamics simulations of target proteins in solutions containing drug‐like probe molecules to characterize their drug‐binding abilities, if any. An important consecutive step is to analyze the trajectories to construct pharmacophore models (PMs) to use for virtual screening of libraries of small molecules. While considerable success has been observed in this type of computer‐aided drug discovery, a systematic tool encompassing multiple steps from druggability simulations to pharmacophore modeling, to identifying hits by virtual screening of libraries of compounds, has been lacking. We address this need here by developing a new tool, Pharmmaker, building on the DruGUI module of our *ProDy* application programming interface. Pharmmaker is composed of a suite of steps: (Step 1) identification of high affinity residues for each probe molecule type; (Step 2) selecting high affinity residues and hot spots in the vicinity of sites identified by DruGUI; (Step 3) ranking of the interactions between high affinity residues and specific probes; (Step 4) obtaining probe binding poses and corresponding protein conformations by collecting top‐ranked snapshots; and (Step 5) using those snapshots for constructing PMs. The PMs are then used as filters for identifying hits in structure‐based virtual screening. Pharmmaker, accessible online at http://prody.csb.pitt.edu/pharmmaker/, can be used in conjunction with other tools available in *ProDy*.

## 1 INTRODUCTION

Drug discovery is a long, costly, and risky process. Computational approaches have been widely used to increase the efficiency of this process and reduce the cost, including quantitative structure–activity relationship (QSAR) studies, docking‐based virtual screening (VS) of libraries of compounds and pharmacophore‐based VS.^1^, ^2^, ^3^, ^4^ QSAR methods evaluate the activities of small molecules in relation to their physicochemical properties using machine learning methods.^5^ Docking simulations evaluate their binding poses and energetics with respect to a target protein and assign scores based on binding affinities.^3^ Pharmacophore models (PMs) define the essential chemical features (such as hydrogen bond donor/acceptor, hydrophobic, aromatic, and charged regions) as well as shared geometric features (e.g., overall volume and shape and relative position of different types of chemical groups) of small molecules that are identified to be pharmacologically effective.^6^

Pharmacophore models can be built by ligand‐ or target‐based methods. In the former case, the PM defines the common patterns of an ensemble of structurally aligned ligands known to have some desirable activity.^7^, ^8^, ^9^, ^10^, ^11^, ^12^ A variety of software, such as HipHop,^13^ HypoGen,^14^ DISCO,^15^ GASP,^16^ PHASE,^17^ and PharmaGist^7^ have been developed to build PMs from ligands. Their performances mainly rely on their ability to handle the flexibility of ligands and their alignment. Ligand‐based approaches have been used in developing inhibitors against Alzheimer's disease,^8^ and targeting topoisomerase I,^9^ 17β‐hydroxysteroid dehydrogenase 2,^10^ and CXC chemokine receptor 2.^11^ Ligand‐based PMs require a set of ligands that are known to have well‐defined pharmacological effects on a target protein or pathway, but the 3D structure of the target(s) is not required.

Target‐based construction of PMs, on the other hand, takes account of the atomic interactions at the putative binding site of the target protein,^18^, ^19^, ^20^, ^21^, ^22^, ^23^ and requires knowledge of the 3D structure of the protein, or at least its ligand‐binding pocket. Methods based on macromolecule‐ligand complex structures, such as LigandScout,^18^ ZINCPharmer,^24^ Pharmit,^25^ and GBPM^26^ can be used to build such PMs. Pharmacophore features are deduced from the geometry and interactions of the ligand bound to the target protein. However, the requirement of a structurally resolved complex with ligand limits the applicability of target‐based pharmacophore modeling.

In view of the existence of structural data for target proteins and their homologs, macromolecule‐based (without ligand) approaches such as GRID,^27^ SuperStar,^28^ HS‐Pharm,^19^ Shaper2,^29^ Pocket V.3,^30^ and CavityPlus^20^ have been developed, which take as input the target structure only, to characterize the binding site. GRID uses an empirical force‐field to evaluate the energy of probes at each grid point around the target structure, and determine the optimal poses at hot spots (positions that exhibit a high propensity to be occupied by ligands).^21^, ^27^ SuperStar learns the distribution of probes from template molecules, and then uses a knowledge‐based method to identify hot spots.^28^ HS‐Pharm identifies hot spots using a machine learning method based on the fingerprints of known ligand‐binding cavities.^19^ CavityPlus^20^ and Pocket V.3^30^ use CAVITY,^31^ a geometry‐based program, to detect cavities and a grid‐based method to define hot spots and assign scores. CavityPlus takes advantage of normal modes predicted by the Gaussian network model (GNM)^31^, ^32^ to evaluate the potential allosteric effects of the druggable sites.^33^ All these tools consider atom–atom interactions and shape complementarity while entropic effects are often overlooked. The conformational entropy, and hence adaptability, of proteins to expose sites that are buried in the resolved structure has been a major motivation for developing flexible docking tools, as opposed to rigid docking.^34^, ^35^, ^36^, ^37^ Yet, another entropic effect, associated with the frequency of binding a site, also plays a dominant role evidenced by the significance of evaluating probe clusters.^24^, ^38^, ^39^ Druggability simulations emerged as an approach that takes account of both types of entropies.

Druggability simulations are simply molecular dynamics (MD) simulations conducted in the presence of a solution containing probe molecules representative of drug‐like fragments, to analyze their binding events onto the “moving” target (protein).^40^, ^41^, ^42^, ^43^, ^44^, ^45^ These simulations demonstrate the ability of proteins to assume alternative conformations, expose potential binding cavities, and selectively bind specific types of probes. Statistical analysis of binding events sheds light onto both enthalpically and entropically favorable hot spots. Enthalpic effects are inferred from the strength/energy of ligand‐protein interaction at the hot spots; entropic effects are deduced from the frequency of binding to a given hot spot. A notable study is that of Carlson and coworkers, where druggability simulations (called Mixed MD cosolvent simulations^46^) were shown to successfully evaluate binding free energies and relative entropies for a series of allosteric proteins.^47^ Furthermore, coarse‐grained models, such as the GNM^31^, ^32^ and the anisotropic network model (ANM) provide unique analytic solutions for the ensemble of conformations sampled under equilibrium conditions,^48^ including potential allosteric changes,^33^ which can be advantageously utilized as input for conducting multiple runs. We have shown that druggability runs of ~40 ns can adequately identify orthosteric and allosteric sites.^12^, ^22^, ^23^, ^42^, ^43^, ^49^ Equally important is the analysis of binding poses and residence times for different probes, which permit us to determine the composition of probes at the hot spots that are most likely to bind drug‐like molecules and estimate the corresponding free energy of binding using simple Boltzmann statistics.^43^ While such analyses have been successfully performed for case studies, such as cytochrome *c*,^22^ γ‐secretase,^49^ ionotropic glutamate receptors (iGluRs),^42^ PTP4A3 phosphatase,^50^ HIV‐1 protease,^51^ K‐Ras,^52^ and several allosteric proteins,^47^ a tool that complements the druggability simulations by systematic analysis of hot spots to construct PMs remains to be established.

Without an easy‐to‐use tool, the preparation of input files for druggability simulations and the analysis of the trajectories to retrieve information for further use in drug discovery requires a great deal of manual operations. Graham et al. developed a PyMOL plugin, Probeview, to facilitate such analyses.^53^ However, the tool does not handle the raw trajectories; instead it takes as input pre‐calculated PDB files that contain occupancy information of grids. We developed DruGUI,^43^ a tool to assist in setting up druggability runs, that is, constructing input files for submitting runs to nanoscale molecular dynamics (NAMD),^54^ and to perform grid‐based analysis of the outputs and their visualization in visual molecular dynamics (VMD).^55^ The Mackerell lab also developed a tool called SILCS‐Pharm for analyzing druggability simulations and extracting pharmacophore features from grid free energy fields called FragMaps.^56^, ^57^ However, there is a need for a suite of tools that would further automate the analysis of trajectories and the characterization of probe‐specific hot spots, to build PMs, and facilitate PM‐based VS. We present here such a tool, Pharmmaker.

We present below the main features of Pharmmaker by way of application to a dimer of the main ligand‐binding domain (LBD) of an AMPA receptor (AMPAR) paralogue GluA2 (PDB 1FTO),^58^ which we recently used in druggability simulations.^42^ AMPARs are glutamate‐gated ion channels that are central to synaptic transmission and plasticity.^59^, ^60^ The LBD binds the neurotransmitter glutamate, leading to conformational changes, initially at the monomer and dimer levels, which trigger receptor activation (opening of the downstream ion channel) and desensitization (entry into a long‐lived agonist‐bound closed channel state).^58^, ^61^, ^62^, ^63^, ^64^, ^65^, ^66^ This domain is also the main binding site for modulators such as cyclothiazide that bind at the dimer interface and block desensitization.^64^, ^67^ The presence of multiple binding sites and the well‐characterized dynamics of this domain^42^ make it suitable for benchmarking and illustrating our methodology.

## 2 RESULTS

### 2.1 Overview of Pharmmaker

**Figure 1:**
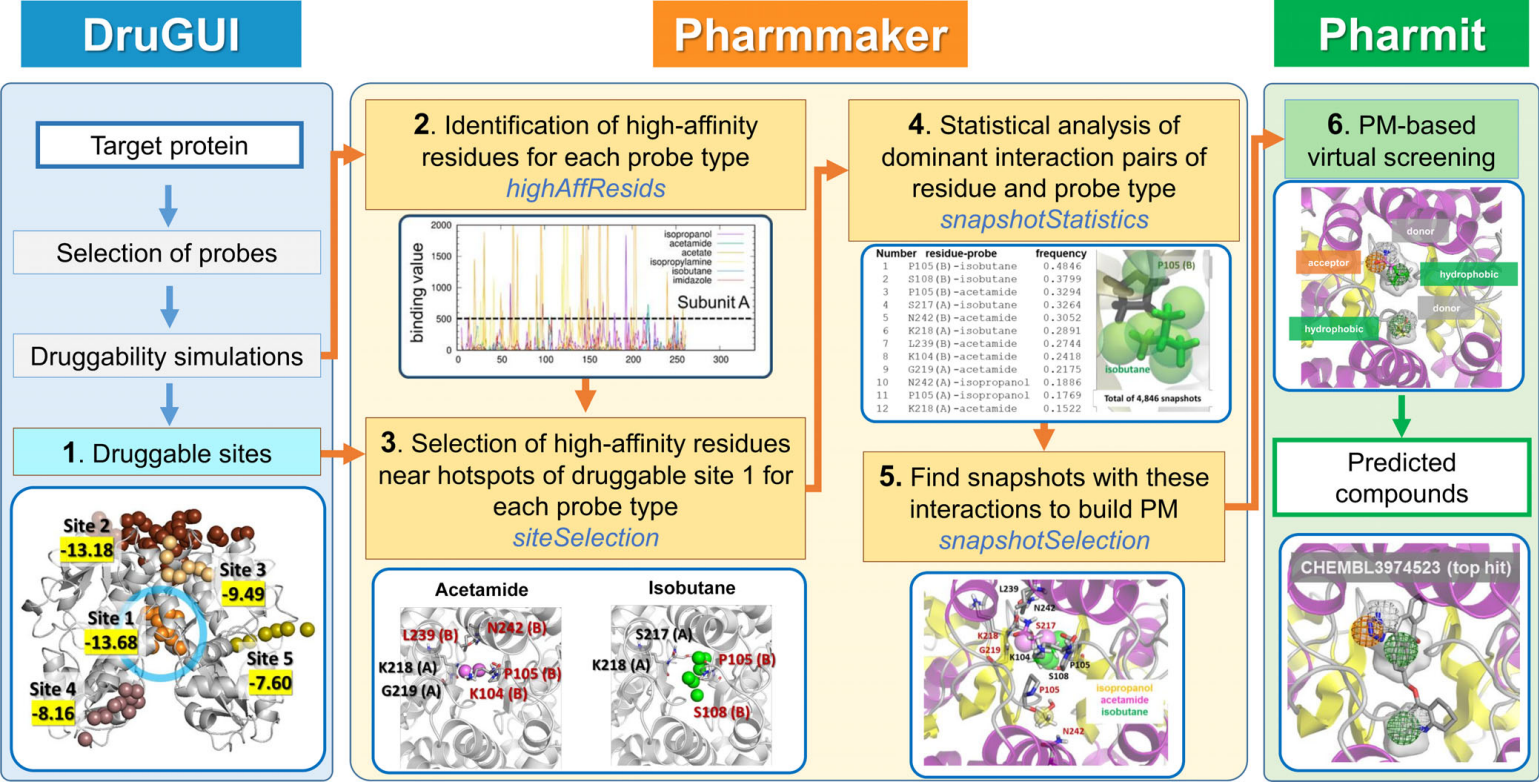
Pharmmaker workflow for constructing pharmacophore models (PMs), in conjunction with druggability simulations (DruGUI) and virtual screening (Pharmit). Pharmmaker uses as input the druggable sites predicted by DruGUI, developed for generating druggability trajectories and identifying druggable hot spots (*blue box*). The output from DruGUI is used by Pharmmaker (Steps 2–5; *yellow box*), to release a PM that is used (in Step 6) for virtual screening (VS) of libraries of compounds using Pharmit (*green box*). See the text for a detailed description of each step

Pharmmaker is a tool with a command‐line interface, which takes outputs from druggability simulations package DruGUI as input, and constructs one or more PMs in a suitable format to be submitted to Pharmit.^43^ Key steps and corresponding outputs are presented below and schematically described in Figure 1. Also presented is a brief description of the preceding druggability analysis using DruGUI, as Step 1. Pharmmaker software and its tutorial can be downloaded from http://prody.csb.pitt.edu/tutorials/pharmmaker/. A more detailed description is also presented in the Supporting Information.

### 2.2 Step 1: Hot spots from druggability simulations

**Figure 2:**
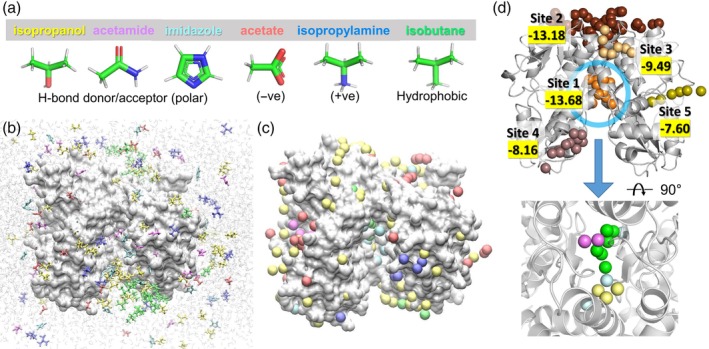
Druggability simulations. (a) The ensemble of probe molecules used in the current study. Six types of probes (isopropanol [*yellow*], acetamide [*magenta*], imidazole [*cyan*], acetate [*red*], isopropylamine [*blue*], and isobutane [*green*]) were used, and their structures and features are indicated at the *bottom*. (b) A snapshot of the simulated system. An LBD dimer of AMPAR subtype GluA2 is shown in *silver surface* representation and probe molecules are shown as sticks colored by types. Water molecules are shown as shaded *light gray* lines in the background. (c) Hot spots from the druggability analysis. Hot spots are voxels in 3D space, which are highly occupied by probe molecules. Clusters of hot spots form druggable sites. Hot spots are obtained for each probe molecule type and are displayed as *balls* in the same color as the probe. (d) Druggable sites revealed by clusters of hot stops. There are five such sites shown in different colors. They are ranked by score (highlighted *in yellow*; comparable to binding energy in kcal/mol) with Site 1 having the highest affinity. Site 1 (*blue* ellipse) is known to bind allosteric modulators that potentiate ion channel currents by blocking desensitization. At the bottom, the zoom‐in view of Site 1 (rotated to show all the hot spots clearly) is shown. We observe hot spots for isopropanol, acetamide, imidazole, and isobutane at Site 1. There are no hot spots for acetate and isopropylamine. AMPAR, AMPA receptor; LBD, ligand‐binding domain

Step 1 is the identification of hot spots from druggability simulations (*blue box* in Figure 1) using the DruGUI module implemented in *ProDy*, as described in our previous studies.^42^, ^43^ In the present illustration, we include six probe molecules: isopropanol, acetamide, imidazole, acetate, isopropylamine, and isobutane (Figure 2a). The number and types of probe molecules can be modified by the user. Figure 2b shows a snapshot from our simulation box containing the target protein and the probe molecules in explicit water. Typically, the concentration of probe molecules is one probe for every 20 water molecules.

Figure 2c shows the results from DruGUI analysis where the spheres display probe‐specific hot spots around the LBD dimer. The hot spots are *color‐coded* as in panel A. Most of the hot spots are on the solvent‐exposed surface of the target as the latter is easily accessible, but we also note a relatively buried site at the interface between the two monomers

Figure 2d shows clusters of hot spots that are highly occupied by probes, which are predicted to serve as druggable sites. There are five druggable binding sites (labeled as Sites 1–5). Their binding energies are obtained using drug‐like combinations of hot spots as described earlier^42^, ^43^ (see Supporting Information). The highest affinity region, Site 1, corresponds to the dimer interface region mentioned above. This site is known to bind allosteric modulators.^67^ We note that this site harbors hot spots for four types of probe molecules, isopropanol, acetamide, imidazole, and isobutane, as shown at the bottom of Figure 2d, meaning that the missing probes, acetate and isopropylamine, do not bind there. In Steps 2–5, we characterize in more detail the specific interactions between the protein residues and the probes to build PMs for Site 1.

### 2.3 Step 2: Identification of residues exhibiting high probe‐specific affinities

**Figure 3:**
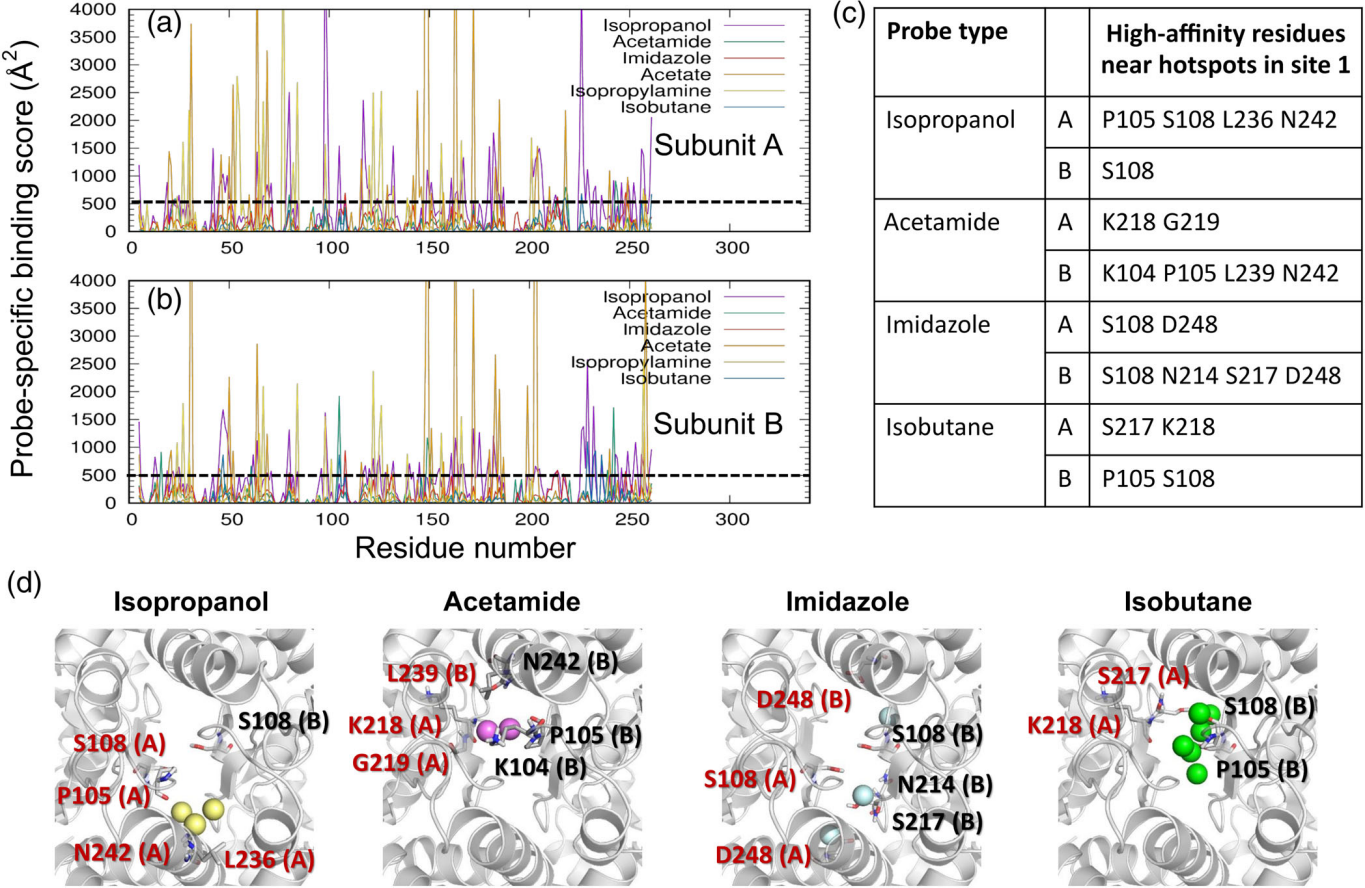
High affinity residues of AMPAR GluA2 LBD dimer interacting with different types of probe molecules. (a, b) Binding score profiles for LBD subunits A (*top*) and B (*bottom*), evaluated for each type of probe molecule (*curves in different colors*, *labeled*). Binding score is as described in the text. Residues with score above 500/Å^2^ (see *dashed line*) are identified as high affinity residues for each type of probe. The residue numbers refer to those in the examined PDB file, corresponding to the isolated LBD. (c) Probe types and high affinity residues at the dimer interface. The corresponding hot spot positions for the probe types and potential coordinating residues are displayed in panel (d). Residues of subunit A are labeled in *red*, and those of chain B, in *black*. AMPAR, AMPA receptor; LBD, ligand‐binding domain

In this step, we identify the residues that are involved in high affinity interactions with probes (Step 2 in Figure 1 and results in Figure 3a,b). To this aim, we assign a probe‐specific binding score to each residue, and generate a binding profile as a function of residue index, for each probe type *p*. Figure 3, panels a and b, illustrate the six profiles, one for each probe, generated for subunits A and B, respectively. The probe‐specific binding score of each residue *i* is defined as *s*(*p*, *i*) = $\sum_{k=1}^{n}{\left(1/d_{\mathrm{ki}}\right)}^{2}$, where *k* is each frame/snapshot index and *n* is the total number of frames recorded during druggability simulations (in our case, 10,000 frames at intervals of 4 ps are recorded for each of the 40 ns runs), *i* is residue index, and *d* is the distance between contact‐making heavy atoms belonging to the respective amino acid *i* and probe *p*. Contact‐making means that they are within close proximity (4 Å) at a given frame *k*. If a given residue‐probe pair exhibits multiple atom‐atom interactions in a snapshot, they are all included in the summation, thus accounting for the tight interaction. The binding score profiles permit us to identify the highest affinity residues by specifying a user‐selected threshold score, above which residues are accepted to exhibit a high affinity for a specific probe. Data analysis shows that 500 Å^−2^ (indicated by the *dashed line* in Figure 3a,b) is a good threshold for 40 ns runs. This analysis can be carried out using the command line program *highaffresid.sh* (see Supporting Information for details).

### 2.4 Step 3: Selection of high‐affinity residues located at druggable sites

The above analysis gives us information on high‐affinity residues for specific types of probes. However, these may be isolated residues, which, in the absence of participation in a cluster of hot spots (or a druggable site) identified by DruGUI, might not stably bind drugs. Hence, we need to select the residues that participate in druggable sites (note that five were identified in Step 1). Therefore, we select from among the high affinity residues identified in Step 2, those that are located at the druggable sites (Step 3 in Figures 1 and 3c,d). Let us consider the highest affinity site (Site 1). This site exhibited high affinity for four different types of probes, isopropanol, acetamide, imidazole, and isobutane, shown in Figure 3c,d. Among the isopropanol‐binding high affinity residues, for example, we select those within 8.0 Å from at least one of the three isopropanol hot spots at this site (*yellow spheres* in Figure 2d, *lower part*, also shown in Figure 3d *left*): P105, S108, L236, and N242 in subunit A and S108 in subunit B. Note that here, we use the residue numbering of the isolated LBD construct in the examined PDB structure,^58^ not that of the full receptor. Repeating the same procedure for each type of probe represented at Site 1, we obtain the four diagrams in Figure 3d, where the hot spots and corresponding high affinity residues are displayed for the four different probe types.

Selection of high affinity residues produces the outputs tabulated in Figure 3c. To generate this type of table in Pharmmaker, the outputs from the previous steps (hot spots from Step 1 and the high affinity residues from Step 2) are used. The high affinity residue files are found automatically, so this analysis can be carried out using the program siteselection.sh (see Supporting Information).

### 2.5 Step 4: Rank‐ordering residue‐probe interactions based on their frequency of occurrence

**Figure 4:**
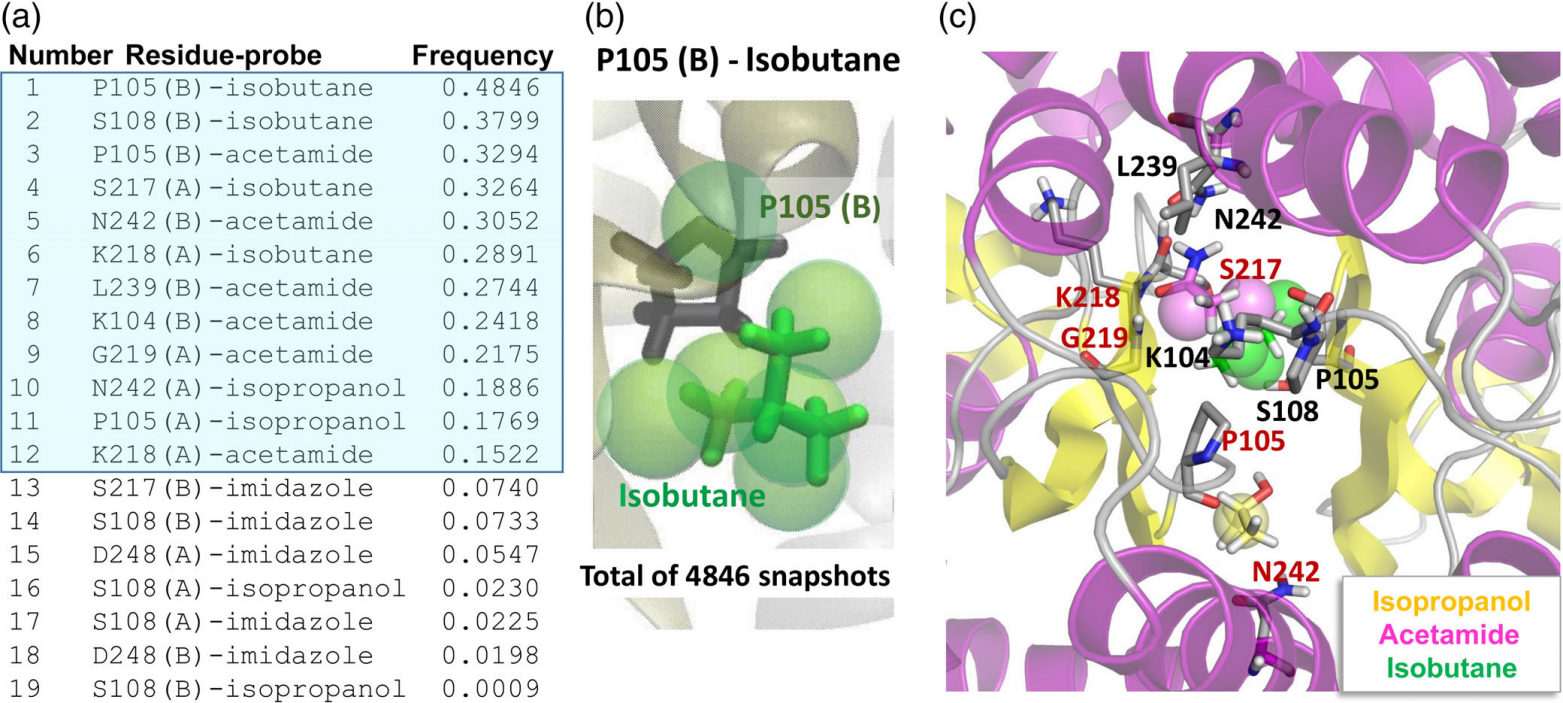
Probe‐residue interaction statistics and most probable interactions. (a, b) Probe‐specific high affinity residues and frequency of specific probe‐amino acid contacts listed in a table (a), and illustrated for one specific case, isobutane near P105 in chain B, in panel b. This residue had one or more contacts with isobutane, distributed over six hot spots (*green spheres*) in a total of 4,846 of 10,000 snapshots (or 48.46% of recorded conformers in our trajectory of 40 ns). The top‐ranking interactions with frequency 0.10 (10%) or higher are highlighted in blue. (c) A snapshot (frame 761) where all the top‐ranking interactions simultaneously take place, used for constructing a PM. Ribbon diagram elements colored *yellow* and *violet* correspond to β‐strands and α‐helices, respectively. Residues of subunit A are labeled in *red*, and those of chain B, in *black*. Also shown are the hot spots with the three probe molecules. PM, pharmacophore model

In this next step (Step 4 in Figures 1 and 4a,b), we focus on the residues selected in Step 3, and rank the corresponding residue‐probe interactions based on their frequency of occurrence (entropy). This is achieved by simply counting the number of snapshots where the specific probe directly interacts with the selected high affinity residues. In the top‐ranking case of P105(B)‐isobutane, for example, there are six isobutane‐specific hot spots (*green spheres* shown in Figures 2d, 3d, and 4b). First, we count the total number of snapshots where an isobutane probe is within 4 Å from the P105(B), based on heavy atoms. Then, we assign them to hot spots whose center is within 1.5 Å from any atom of the probe. We count the number of snapshots with a probe near this residue and occupying any of these six hot spots as shown in Figure 4b, which is 4,846 in this case, out of 10,000 snapshots per run of 40 ns, yielding a frequency of 0.4846. We repeat the procedure for each selected residue‐probe pair. The resulting frequencies are rank‐ordered and listed in Figure 4a. This analysis can be carried out using the program snapshotstatistics.sh, which takes the output from previous steps as inputs (see Supporting Information).

### 2.6 Step 5: Construction of a pharmacophore model

**Figure 5:**
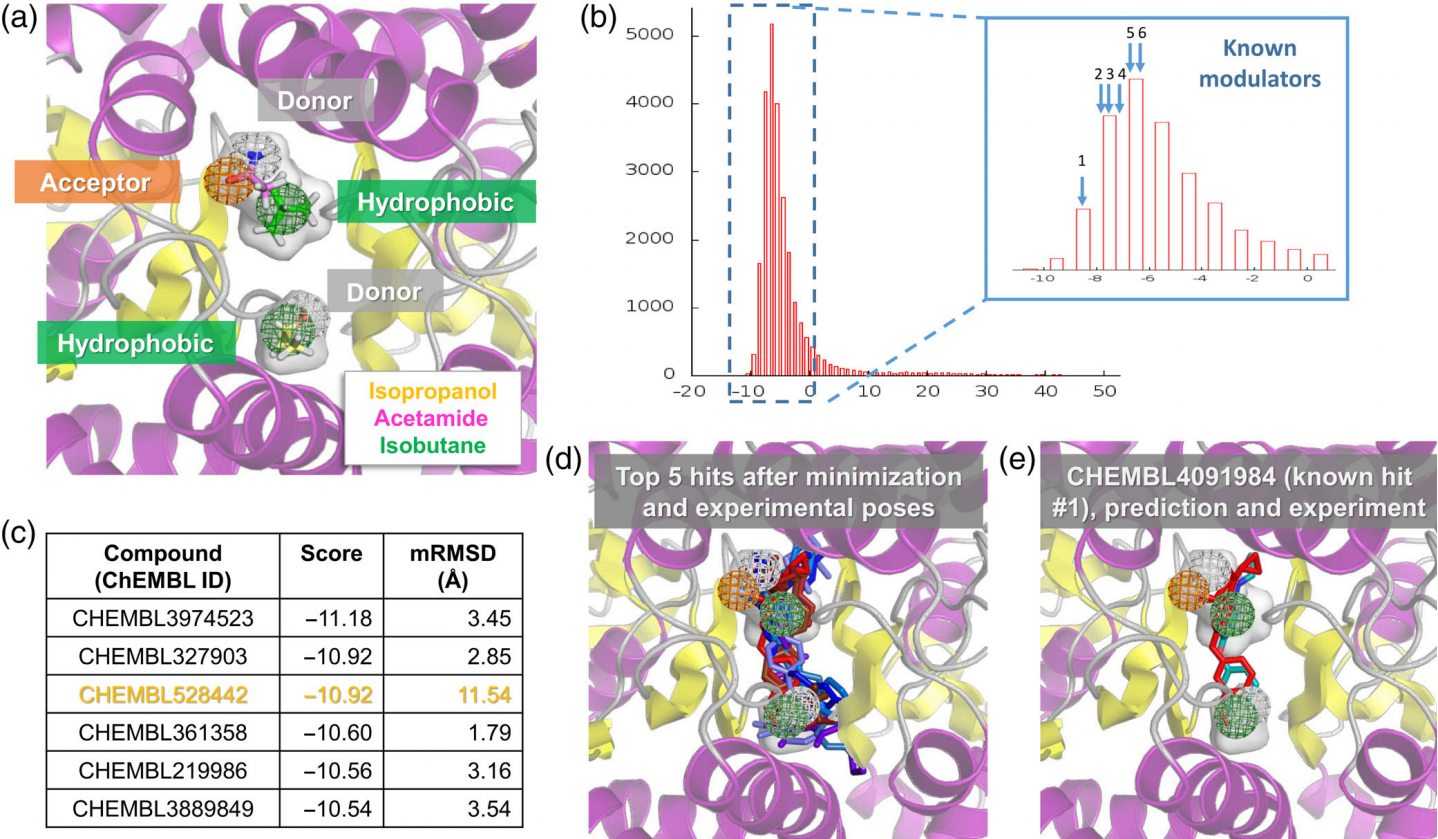
Compounds obtained from virtual screening of our PM against the ChEMBL 25 library of small molecules. (a) PM constructed based on the snapshot displayed in Figure 4c, using the binding position and orientation of three probe molecules: acetamide, isobutane, and isopropanol. C atoms are colored by probe as in previous figures, H, N, and O atoms are colored *white*, *blue*, and *red*, respectively. Hydrogen bond donor (*gray*) and acceptor (*orange*) features were used for acetamide (both donor and acceptor) and isopropanol (donor), and hydrophobic features (*green*) were used for isopropanol and isobutane. Selected probes span the *gray transparent* volume, which is displayed in other panels too for comparison. (b) Histogram showing AutoDock Vina scores from querying the ChEMBL 25 database with Pharmit. The majority of scores are negative (corresponding to favorable binding energies). The inset enlarges this region with negative scores and indicates the scores for six known AMPAR modulators listed in ChEMBL 25 (see Table 1). (c) AutoDock Vina score and minimization RMSD (mRMSD) between docked compounds and the input pharmacophore features are listed for the top hits (compounds with the highest binding affinity or most negative scores).One of the compounds (highlighted in orange) has a high mRMSD value, meaning its preferred binding pose after minimization is not consistent with the input PM. (d). Optimized binding poses of the top five hits shown in various shades of *blue*, along with the experimentally resolved modulators (R,R)‐2a, (R,R)‐2b, and 11m from PDB structures 3BBR, 4U5B, and 5OEW in *red*. (e) The predicted binding pose of the top known modulator (11m) and its experimental pose show good agreement with the PM. See also Figure S3 for additional hits and experimentally determined compounds (Table 1) that match the PM. PDB, protein data bank; PM, pharmacophore model

This is the final step in Pharmmaker (Step 5 in Figures 1, 4c, and 5a). We extract the snapshots that exhibit the most frequent interactions (the top 12 in Figure 4a using the frequency of occurrence 10% as default cutoff). We found a total of 13 snapshots that include these top 12 interactions. That is, all 13 snapshots display isobutane interacting with the residues P105(B), S108(B), S217(A), and K218(A), acetamide interacting with P105(B), N242(B), L239(B), K104(B), G219(A), and K218(B), and isopropanol interacting with N242(A) and P105(A). While the interactions could be made by different probes of the same type, it is also possible for only one probe to be interacting with multiple residues at the same time.

Figure 4c displays one such conformation representing a highly robust network of interactions, with side chains oriented to achieve optimal probe binding. The 13 snapshots/conformers that jointly display all the top‐ranking interactions are indeed quite similar structurally, with an average RMSD of 1.9 Å (see Figure S1a). These conformers are used as inputs for structure‐based virtual screening in the next step. The use of probe poses and corresponding target conformations from the same snapshots allows us to obtain more accurate results. A specific target conformation is important in order to have accurate binding scores in virtual screening, for example, probe position and affinity are dependent on the side chains' rotameric states as well as the fluctuations in the backbone. This analysis can be carried out using the program snapshotstatistics.sh, which it takes the output from previous steps as inputs (see Supporting Information).

We use each of the snapshots selected in this step to construct a PM, as illustrated for one of the snapshots (numbered 761) in Figure 5a. The PM consists of two hydrophobic groups, one hydrogen bond acceptor and two hydrogen bond donors, arranged in a well‐defined geometry, occupying a cavity (shown by the *semi‐transparent gray volume*) at the interface between the two LBD protomers. It was created by selecting appropriate features based on the dominant interactions, which would then be realized in the next step.

### 2.7 Step 6: Virtual screening of libraries of compounds using the PM

Figure 5 (Step 6, *green box* in Figure 1) shows the results from screening our PM against the ChEMBL database^68^, ^69^ using the Pharmit Server.^25^ PM features were treated as spheres of 1 Å radius for matching against database compounds, and hits were filtered to only include 1 entry per compound and exclude compounds larger than 500 Da. This was then succeeded by minimization docking using AutoDock Vina^70^, ^71^ within Pharmit, yielding the binding scores and hits shown in Figure 5b,c and Table 1 for snapshot 761. Other snapshots gave similar but distinct results as shown in Figure S1b, S2, and Table S1. We therefore recommend users to use all the snapshots containing the most dominant interactions and compare them. While the global interactions are maintained throughout the simulation, the exact conformation will likely change over time, allowing them to sample more possible binders. It should, however, be noted that after a certain degree of conformational change, DruGUI analysis results may not be meaningful when carried out over a whole trajectory, which is why we use 40 ns.

**Table 1:** Compounds with experimental verification, identified in ChEMBL (release 25)

Compound (ChEMBL ID)	pEC_50_ a	Initial RMSD (Å)	Binding energy (kcal/mol)	RMSD after minimization (mRMSD; Å)	Rank among all compounds^b^	Rank among known compounds^b^
CHEMBL4091984	8.700^72^	0.57	−8.34	1.63	1,159	1
CHEMBL1214203	5.100^80^	0.56	−7.70	2.75	2,957	2
CHEMBL4060993	7.336^72^	0.65	−7.44	1.21	4,001	3
CHEMBL1290503	5.398^81^	0.60	−6.97	2.77	6,297	4
CHEMBL1277180	5.796^82^	0.70	−6.72	2.30	7,645	5
CHEMBL1214334	5.900^80^	0.75	−6.57	2.26	8,452	6

a Extracted from the cited articles, or calculated as −log_10_(EC_50_).

b After minimization.

Figure 5c lists the top‐ranking compounds (hits) from VS, and the corresponding AutoDock Vina scores (or binding energies) and RMSDs with respect to the pharmacophore features after energy minimization (mRMSD). We note that a score below −10 kcal/mol represents a highly favorable (nanomolar range) interaction. The poses of the top five compounds are shown in *blue sticks* in Figures 5d and S3a, along with the poses of known allosteric modulators in red, a benzothiadiazine, designated as compound 11m (PDB id: 5OEW^72^; also shown in Figure 5e), and the biarylpropylsulfonamides (R,R)‐2a (PDB id: 3BBR),^73^ and (R,R)‐2b (PDB id: 4U5B)^74^ (see Figure S3b for individual examples). Therefore, the hits occupy the same space as the known allosteric modulator, and exhibit similar features as illustrated in panels d and e of Figure 5. It remains to be experimentally tested and verified whether these compounds could function as well or even better than the existing modulators

The screening against ChEMBL also allowed us to identify some experimentally verified compounds with good scores (see Figure 5b,e, S3c, Table 1). The best one (first row of Table 1) corresponds to the compound 11m mentioned above and the predicted binding pose matches the known one in the resolved structure (PDB id: 5OEW) very well (Figure 5e). Interestingly, these compounds show small RMSDs (< 1.0 Å) with respect to the PM before minimization docking (Table 1) and, in some cases, the binding scores after docking correlate very well with the inferred binding affinity from functional experiments (pEC_50_ in Table 1). The compound 11m is likely the most potent AMPAR modulator to date with an effective concentration for 50% activity (EC_50_) of 2.0 nM,^72^ corresponding to a pEC_50_ (− log_10_ EC_50_) of 8.700 in line with the binding score of −8.34 kcal/mol. Another compound in the same series (known compound #3; Figure S3c) has a pEC_50_ of 7.336 and a binding score of −7.44 kcal/mol. Therefore, the new compounds predicted by our method have the potential to bind with sub‐nanomolar affinity.

## 3 CONCLUSION

Structure‐based VS is not easy, especially if there is no information about binding pocket, binding features, and poses; and target flexibility makes it an even more challenging problem. A strong aspect of our method is that it uses multiple target conformations dependent on the binding poses of probes where they interact during druggability simulations. Therefore, the binding score in VS can be more evaluated in a more realistic manner. Also, we can have multiple PMs for the same site, with different target conformations and probe poses, which can be analyzed statistically. Furthermore, multiple different compositions of PM features can be explored. In this article, we focused on the highest affinity site, which is a known allosteric site, but we can focus on other sites that harbor clusters of hot spots and on specific residues if necessary. Our method is purely computational and unbiased, and we believe that this new tool will assist in current efforts in drug discovery and development, especially in the identification of allosteric modulators.

## 4 METHODS

The manuscript is a tool description overall. So, we present below a brief overview only, and more details including all intermediate steps, commands, and quantitative data, are provided in the Supporting Information and in the Tutorial accessible online.

Druggability simulations and trajectory analyses were performed as described previously.^42^, ^43^ Briefly, simulations were run using the probe set shown in Figure 2 (selected as representatives of drug fragments with different physicochemical properties) using the molecular dynamics (MD) simulation package NAMD^54^ with the CHARMM22 force field for proteins,^75^ the TIP3P water model,^76^ and the CGenFF force field^77^ (version 43) for the probes. Trajectory analyses were performed using the DruGUI module^43^ of *ProDy*.^48^, ^78^

The target protein used for illustration of Pharmmaker is AMPAR GluA2 LBD dimer (PDB id: 1FTO).^58^ Two independent runs were performed for AMPAR GluA2 LBD dimer, which yielded similar results in DruGUI analysis, in agreement with experiments.^58^ We use one of them here for illustrative purposes. The latter presented five druggable sites. We focused on the highest affinity site indicated by DruGUI analysis, which agreed well with experiments.^58^ All MD snapshots were superposed onto the reference PDB structure using C^α^‐atoms and a cubic grid‐based representation of the space was used for the analysis. Grid edge size was set to 0.5 Å. Probe molecules having non‐hydrogen atoms within 2.5 Å from protein atoms were considered to interact with the protein. For each probe type, the individual occupancy of grids was calculated using their centroids. We evaluated the occupancy of each probe for a given voxel. High occupancy voxels, called hot spots, within a distance less than 5.5 Å were merged and druggable sites were defined by clusters of at least six such hot spots. We obtained five druggable sites as shown in Figure 2d; details of binding affinity calculations are explained in the Supporting Information. The druggable sites were analyzed further to build a PM with our new tool called Pharmmaker, written in Tcl and Bash. This tool is described in our results section and outlined in Figure 1. Details of each step are described in the Supporting Information, and the tutorial files are accessible online at http://prody.csb.pitt.edu/tutorials/pharmmaker.

We used Pharmit, which is for VS of large compound databases using pharmacophore features, molecular shape, and energy minimization.^25^ We applied the following filters: 1 hit per molecule and molecular weight ≤ 500. Features as described in the results were used for screening the ChEMBL database.^68^ Data visualization are performed using ProDy 1.10.10,^78^ VMD 1.9.1,^55^ and PyMOL 1.8.6.^79^

## Supporting information


**Appendix S1.** Supporting Information.
