# Supplementary material for: Pharmmaker: Pharmacophore modeling and hit identification based on druggability simulations
Source: Protein Sci. 2019 Dec 4;29(1):76–86. doi: 10.1002/pro.3732 (PMC6933858; doi:10.1002/pro.3732)
Supplement: Supplementary file 1 — Appendix S1. Supporting Information. [file PRO-29-76-s001.docx]

**Supplemental** **Material**

This material includes Supplemental Methods, and Supplemental Tables and Figures.

**Supplemental Methods**

***Details for the process of using Pharmmaker***

Based on druggability simulations, we find dominant interactions between probe molecules and residues of a target protein, collect snapshots from the trajectory with the dominant interactions, and build pharmacophore model from the snapshots. The files used in this example can be downloaded from <http://prody.csb.pitt.edu/tutorials/pharmmaker>, which also provides more details of alternative commands. The results of a more in-depth analysis of these druggability simulations was published in a recent paper.^1^

**Step 1: Hot spots from druggability simulations**

Step 1 is the identification of druggable sites and hot spots using druggability simulations. The running and analysis of such simulations is carried out using DruGUI^2^, VMD^3^, and NAMD^4^. The various outputs of used by Pharmmaker are shown in **Fig 2** of the main text. Pharmmaker uses both the druggability simulations themselves (in steps 2, 4 and 5) and the druggable sites and hot spots (in step 3).

The setup and analysis was also performed in a fully automated way using the DruGUI module of ProDy/VMD by localizing the probes on a three-dimensional grid (with spacing 0.5 Å) and quantifying binding energies with the inverse Boltzmann relation as described earlier^2^. Briefly, each probe molecule was localized to a particular grid cell using the centroid of each molecule based on heavy atoms. The ratio of the actual occupancy of each probe in each grid cell on the protein surface (n_i_) relative to that in solution (n_0_) was used to calculate the binding free energy using ∆G_i_ = -RT ln(n_i_/n_0_) where R is the gas constant and T is the absolute temperature (in K). The resulting binding free energy map identifies interaction spots with low energy for one or more probe types, which we call hot spots. Druggable sites were identified as clusters of hot spots and their maximal binding affinities were calculated as the sum of binding energies of their hot spots, as described previously, allowing us to rank the druggable sites and select site 1 as the highest affinity region.

The hot spots in site and the probe types located at them are provided to Pharmmaker in a PDB file. We analyze site 1, which is the highest affinity region, using the hot spots in the PDB file shown below.

=========== *dg_site_1.pdb* ===========

ATOM 1 C2 IBTNA 1 -0.244 0.481 -1.257 0.86 -2.31 C

ATOM 2 C2 ACAMA 2 0.756 1.981 -5.757 0.98 -2.30 C

ATOM 3 C IMIDA 3 -1.744 -4.519 -0.257 0.49 -2.07 C

ATOM 4 C2 IPROA 4 -1.744 -8.519 -3.757 1.00 -1.97 C

ATOM 5 C2 IPROA 5 -0.244 -6.519 0.743 0.50 -1.70 C

ATOM 6 C2 IBTNA 6 0.756 0.481 -3.757 0.72 -1.67 C

ATOM 7 C2 IBTNA 7 -1.244 2.981 -1.757 0.67 -1.66 C

ATOM 8 C2 IPROA 8 -3.744 -6.519 -3.257 0.97 -1.61 C

ATOM 9 C IMIDA 9 -4.244 5.981 1.243 0.80 -1.58 C

ATOM 10 C2 IBTNA 10 -0.744 3.981 -5.757 0.54 -1.48 C

ATOM 11 C IMIDA 11 1.756 -9.019 4.243 0.98 -1.31 C

ATOM 12 C2 IBTNA 12 -1.244 -2.019 -0.757 0.72 -1.23 C

ATOM 13 C2 IBTNA 13 -2.244 5.481 -0.757 0.72 -1.13 C

ATOM 14 C2 ACAMA 14 3.256 1.481 -5.757 0.99 -1.03 C

=========== *dg_site_1.pdb* ===========

**Step 2: Identification of residues exhibiting the highest probe-specific affinities**

In this step, we identify the residues having high affinity interactions with each probe (see **Fig 3** in the main text). This requires paths to files containing the initial structure and the trajectory (in our case in PDB and DCD format), which are outputs of step1. Most other inputs to the program can be calculated from these files but there is also an option to provide custom values.

This program can be used in two ways:

1. Calculate binding value for all residues based on one or more druggability simulations
2. Select high affinity residues by applying a cutoff to binding values calculated previously

***Step 2a: Calculation of probe-specific binding values***

For the first step, we use the following command:

*highaffresid.sh 0 ../drugui-simulation/protein-probe.pdb ../drugui-simulation/protein-probe.dcd*

In this command, we provide a zero at the beginning to indicate we are not using a binding value cutoff and then provide a starting structure and trajectory file for the simulation(s) to be analysed.

Input arguments are provided after the program name in the following order with optional values in grey.

| **Description** | **Default value** |
| --- | --- |
| ***Binding value cutoff (Å^-2^) or lack therefore***  We can provide a zero in this step. Other values can be provided instead of zero (see next step). **Some value must be provided for the program to work.** | ‘’ |
| ***Starting structure for druggability simulation***  The path to one file (for all trajectories) or as many files as trajectories separated by commas. | ‘’ |
| ***Trajectory for druggability simulation***  The path to one file or multiple files separated by commas. Likewise, we use DCD files. | ‘’ |
| ***Chains to include in the analysis***  The word **all** or a list of chain IDs separated by commas. | all |
| ***Probes to include in the analysis***  The word **all** or a list of 4-letter probe names separated by commas. | all |
| ***First residue ID***  The word **first** or a residue ID for the first residue to include from each chain. A single value can be provided for all chains, or values can be provided for each chain. | first |
| ***Last residue ID***  The word **last** or a residue ID for the first residue to include from each chain. A single value can be provided for all chains, or values can be provided for each chain. | last |
| ***First frame number***  The word **first** or frame numbers for the first frames to include in the analysis. One value can be provided for all trajectories, or values can be provided for each trajectory. | first |
| ***Last frame number***  The word **last** or frame numbers for the first frames to include in the analysis. One value can be provided for all trajectories, or values can be provided for each trajectory. | last |

The arguments must be separated by spaces and may not contain spaces themselves. To provide multiple values to an argument, use commas as separators instead.

The output includes a directory ***highaffresid*** containing a file for each combination of chain ID and probe type as illustrated below. The first column is residue number, and the second column is binding value for chain A and probe type IPRO (as an example) as described in the manuscript. By plotting the data in such files for all the probes, we obtain graphs as in **Fig 3A and 3B** of the main text.

=========== *A-IPRO.dat* ===========

5 1196.53

6 270.58

7 94.2

8 0.00

9 0.00

~~

=========== *A-IPRO.dat* ===========

***Step 2b: Selection of high affinity residues with high probe-specific binding values***

From this data/graph, we select a binding value cutoff, which we use to select high affinity residues. These are residues with a binding value larger than the cutoff value. In our case we select ***500***.

To apply this cutoff, we run the following command:

*highaffresid.sh 500*

The only value we provide is the cutoff as described below. By not providing structure and trajectory files, we tell the program to use results calculated previously.

| **Description** | **Default value** |
| --- | --- |
| ***Binding value cutoff (Å^-2^)***  A binding value cutoff above which residues are selected as high affinity residues. The binding value is essentially an inverse square distance potential term for each interaction within a cutoff of 4 Å. | ‘’ |

The high affinity residues for a particular chain and probe are listed in a second set of files, as illustrated below for chain A and IPRO. These files are needed for the step 3.

=========== *A-IPRO**-highaffresid.dat* ===========

5 19 22 25 29 42 45 46 47 48 49 50 51 64 67 68 69 ~~

=========== *A-IPRO-highaffresid.dat* ===========

We also obtain a summary file *highaffresid.dat*, which provides an easier way to see all the high affinity residues at once:

=========== *highaffresid.dat* ===========

ACAM A 23 24 80 213 218 219 243 244

ACTT A 20 21 31 45 46 50 52 60 64 69 ~~

~~

=========== *highaffresid.dat* ===========

Other files are also created that are used by later steps and discussed below.

**Step 3. Selection of high-affinity residues in site 1**

This is a simple step, which selects high affinity residues and hot spots near each other (within a user-defined cutoff). Here we choose the hot spots in site 1, which is the highest affinity region based on druggability simulations. The output from this step is shown in **Fig 3C and D**.

To perform this analysis, we run the following command:

*siteselection.sh ../drugui-analysis/dg_site_1.pdb*

A more custom command could be given that provides more input arguments but most of the values are taken from the files written by the previous step (coloured in grey below). The arguments for this command should be given in the following order.

| **Description** | **Default value** |
| --- | --- |
| ***Hot spots file(s)***  One hotspots.pdb file should be provided. This can be all hot spots from druggability analysis or the ones in a particular druggable site. **This value is required and the program cannot run without it.** | ‘’ |
| ***A cutoff for selecting high affinity residues and hot spots (Å)***  Residues and hot spots within this distance of eah other will be included in regions of interest. | 8 |
| ***High affinity residues file(s)***  One or more files ending in highaffresid.dat or a directory containing them. These should not by highaffresid.dat itself but all the probe- and chain-specific ones that just contain numbers. | highaffresid |
| ***Starting structure for druggability simulation***  The path to a file. If this file is a PDB file, it will be used directly. Otherwise, the program will read through the file to find a list of files and take the first one. The default behaviour is to use **struc-list.dat**, which is generated in the previous step. It lists all structure files used by *highaffresid.sh* | struc-list.dat |
| ***Chains to include in the analysis***  A list of chain IDs or a file containing them. The default behaviour is to use **chain-list.dat**, which is generated in the previous step. It lists all chains used by *highaffresid.sh* | chain-list.dat |
| ***Probes to include in the analysis***  A list of chain names or a file containing them. The default behaviour is to use **probe-list.dat**, which is generated in the previous step. It lists all probes used by *highaffresid.sh* | probe-list.dat |
| ***First frame number***  The word **first** or frame numbers for the first frames to include in the analysis. One value can be provided for all trajectories, or values can be provided for each trajectory. | first |
| ***Last frame number***  The word **last** or frame numbers for the first frames to include in the analysis. One value can be provided for all trajectories, or values can be provided for each trajectory. | last |

The program creates a new directory ***dg_site_1*** in the directory ***highaffresid***.

It contains two files. *hotspots2.pdb* contains the set of hot spots from the input that are within the cutoff of the high affinity residues. In our case, this matches the input *dg_site_1.pdb* but this may not be the case.

*highaffresid2.dat* has the same format as *highaffresid.dat* above but has fewer residues:

=========== *highaffresid2.dat* ===========

ACAM A 218 219

IBTN A 217 218

IMID A 108 248

IPRO A 105 108 236 242

ACAM B 104 105 239 242

IBTN B 105 108

IMID B 108 214 217 248

IPRO B 108

=========== *highaffresid2.dat* ===========

**Step 4. Ranking dominant interactions between pairs of residues and probes**

In this step, we count snapshots that have probes near high affinity residues and located in the hot spots. The inputs are high affinity residues and hot spots files (output by step 3). Here, we simply use the command below. All parameters are set using default values for inputs.

*snapshotstatistics.sh*

The parameters you can give are as follows with all of them being optional ones in grey:

| **Description** | **Default value** |
| --- | --- |
| ***Input directory for results from site selection step (step 3)***  One or more directories containing inputs (highaffresids2.dat and hotspots2.pdb as described above) or a file pointing to them. The default file is generated by step 3. | site-list2.dat |
| ***Residue-probe interaction cutoff (Å)***  Residue-probe interactions within this cutoff are counted. | 4 |
| ***Probe-hotspot cutoff (Å)***  Probes are included if they are within this distance of hot spots | 1.5 |
| ***Trajectories for druggability simulations***  The path to a file. If this file is a DCD file, it will be used directly. Otherwise, the program will read through the file to find a list of files and take the first one. The default behaviour is to use  **traj-list.dat**, which is generated in the previous step. It lists all structure files used by *highaffresid.sh* | traj-list.dat |
| ***Starting structures for druggability simulations***  The path to a file. If this file is a PDB file, it will be used directly. The default behaviour is as above but using **struc-list.dat** | struc-list.dat |
| ***Chains to include in the analysis***  A list of chain IDs or a file containing them. The default behaviour is to use **chain-list.dat**, which is generated in the previous step. It lists all chains used by *highaffresid.sh* | chain-list.dat |
| ***Probes to include in the analysis***  A list of chain names or a file containing them. The default behaviour is to use **probe-list.dat**, which is generated in the previous step. It lists all probes used by *highaffresid.sh* | probe-list.dat |
| ***First frame number***  The word **first** or frame numbers for the first frames to include in the analysis. One value can be provided for all trajectories, or values can be provided for each trajectory. | first |
| ***Last frame number***  The word **last** or frame numbers for the first frames to include in the analysis. One value can be provided for all trajectories, or values can be provided for each trajectory. | last |

After running the script, an output directory is created called ***snapshot*** if it doesn’t already exist. Inside this directory will be a directory called ***dg_site_1*** and this directory will contain outputs for this site.

The most important output file is *zlist-count* shown below:

=========== *zlist-count* ===========

4846 snapshot-s1/z.B.105.IBTN/outfr.dat

3799 snapshot-s1/z.B.108.IBTN/outfr.dat

3294 snapshot-s1/z.B.105.ACAM/outfr.dat

~~~

=========== *zlist-count* ===========

This shows how many snapshots (highlighted in *yellow*) include each interaction (highlighted in *cyan*).

We also obtain a file *zlist-frequency* where the counts are divided by the number of frames (see **Fig 4A**).

**Step 5: Construction of a pharmacophore model**

In this step, we collect snapshots which have dominant interaction pairs. We know dominant interaction pairs from the previous step, and we use a cutoff of 1000 to select the top ones based on their frequency. This selects the top 12 interactions in our case.

We use the command below:

*snapselection.sh*

The parameters are as follows:

| **Description** | **Default value** |
| --- | --- |
| ***Cutoff frequency or count for dominant interactions considered when selecting snapshots***  Dominant interactions are scored by how frequent they are throughout the trajectory in the previous step. We provide a cutoff that can be either a frequency (decimal below 1) or a count (above 1). The default value is 0.1, which is 10 % | 0.1 |
| ***Input directory for results from site selection step (step 3)***  One or more directories containing inputs from the previous step or a file pointing to them. We process it to get the last part, which was used in step 4. | site-list2.dat |
| ***Trajectories for druggability simulations***  The path to a file. If this file is a DCD file, it will be used directly. Otherwise, the program will read through the file to find a list of files and take the first one. The default behaviour is to use **traj-list.dat**, which is generated in the previous step. It lists all structure files used by *highaffresid.sh* | traj-list.dat |
| ***Starting structures for druggability simulations***  The path to a file. If this file is a PDB file, it will be used directly. Otherwise, the program will read through the file to find a list of files and take the first one. The default behaviour is to use **struc-list.dat**, which is generated in the previous step. It lists all structure files used by *highaffresid.sh* | struc-list.dat |
| ***First frame number***  The word **first** or frame numbers for the first frames to include in the analysis. One value can be provided for all trajectories, or values can be provided for each trajectory. | first |
| ***Last frame number***  The word **last** or frame numbers for the first frames to include in the analysis. One value can be provided for all trajectories, or values can be provided for each trajectory. | last |

After performing this analysis, output files are target and probe coordinates of snapshots in PDB format, including the top 12 interactions. A total of 13 snapshots have all 12 interactions. Using the coordinates of the probes, we can define binding features and build a pharmacophore model (PM) for each snapshot.

**Step 6:** **Virtual screening of libraries of compounds using the PM**

In this step, we perform virtual screening using Pharmit server.^5^ The output files of step 5 (PDB files of target and probes) are used as inputs for the server. The majority of pharmacophore features were used with the exception of redundant hydrogen bond donors and acceptors at the same location that caused the screening to have a very small number of results. Hydrogen bond donor and acceptor features were also modified to have a size of 1 Å.

**Supplemental Table**

**Supplementary Table S1: Overlaps between compounds identified when using different snapshots for pharmacophore model generation with the same features.**

|  | 761 | 1080 | 1223 | 1244 | 1325 | 1449 | 1453 | 1461 | 1712 | 1801 | 2391 | 4453 | 6402 |
| --- | --- | --- | --- | --- | --- | --- | --- | --- | --- | --- | --- | --- | --- |
| 761 | 100% | 3% | 3% | 3% | 5% | 15% | 10% | 12% | 10% | 7% | 5% | 20% | 7% |
| 1080 | 20% | 100% | 54% | 24% | 19% | 16% | 15% | 16% | 19% | 16% | 31% | 31% | 27% |
| 1223 | 19% | 50% | 100% | 28% | 13% | 14% | 12% | 14% | 15% | 15% | 30% | 28% | 33% |
| 1244 | 16% | 17% | 21% | 100% | 7% | 15% | 12% | 13% | 9% | 10% | 47% | 27% | 31% |
| 1325 | 23% | 13% | 10% | 7% | 100% | 22% | 27% | 23% | 47% | 9% | 10% | 13% | 11% |
| 1449 | 33% | 5% | 5% | 7% | 10% | 100% | 55% | 53% | 34% | 8% | 10% | 17% | 10% |
| 1453 | 24% | 5% | 4% | 6% | 13% | 61% | 100% | 64% | 48% | 6% | 9% | 13% | 8% |
| 1461 | 26% | 5% | 5% | 6% | 10% | 54% | 58% | 100% | 31% | 6% | 9% | 14% | 9% |
| 1712 | 22% | 6% | 5% | 4% | 21% | 35% | 44% | 31% | 100% | 7% | 7% | 10% | 8% |
| 1801 | 32% | 10% | 10% | 9% | 8% | 16% | 11% | 12% | 14% | 100% | 11% | 20% | 19% |
| 2391 | 19% | 15% | 16% | 33% | 7% | 17% | 14% | 15% | 11% | 9% | 100% | 31% | 29% |
| 4453 | 37% | 8% | 7% | 9% | 5% | 14% | 10% | 12% | 8% | 8% | 16% | 100% | 15% |
| 6402 | 22% | 11% | 15% | 18% | 6% | 14% | 11% | 12% | 11% | 12% | 25% | 26% | 100% |

The matrix is normalized by dividing the rows by their diagonal values, which gives an asymmetric matrix. Each element *ij* from row *i* and column *j* is the fraction of all the compounds identified using the snapshot number for row *i* that are also seen in the set identified using the snapshot number for column *j*. See Supplementary Figure S2.

**Supplemental Figures**

**Figure S1.** **Similarity between protein structures and compounds identified for different snapshots.** **A.** The structural difference between protein conformations in the different snapshots indicated by the root mean square deviation (RMSD) in atom positions for all heavy atoms including side chains. Values below 3 Å indicate that the fold remains similar but fluctuations in the structure are evident with divergences from original conformer occurring gradually over time. **B.** The similarity between the compounds identified is shown using the fractional overlaps. This matrix is normalized by dividing the rows by their diagonal values, which gives an asymmetric matrix. Each element ij is the fraction of all the compounds identified using the snapshot number for row i that are also seen in the set identified using the snapshot number for column j. See **Supplementary** **Table S1** for percentages.

**Figure S2. Three-way Venn diagrams comparing compounds obtained from virtual screening of the same PM from different snapshots against the ChEMBL25 library of small molecules**. Each panel shows the overlap between three sequential snapshots that have the top 12 most stable interactions. The last snapshot from each panel (number circled in *red* and *blue* Venn circle) is shown again in the next panel (number circle in *blue* and *red* Venn circle).


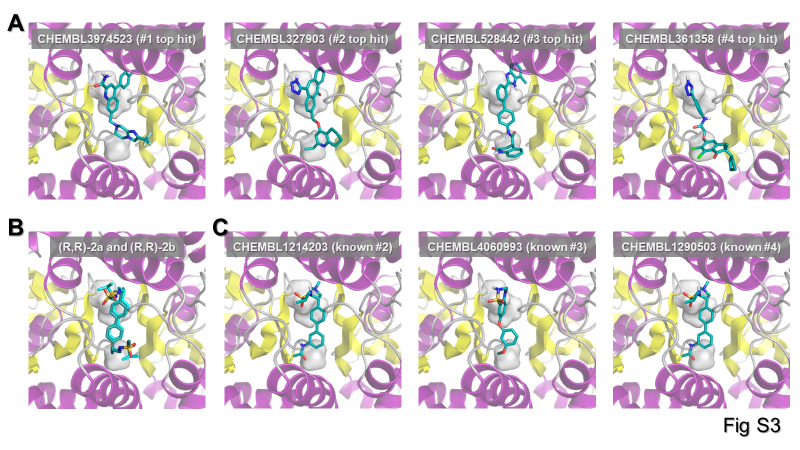


**Figure S3.** **More details about compounds obtained from virtual screening of our PM against ChEMBL library of small molecules (related to Fig 5C-E).** **A.** Each of the top 5 hits is shown individually along with the pharmacophore model. **B.** The experimental compound poses for the closely related known compounds (R,R)-2a and (R,R)-2b in PDB structures 3BBR and 4U5D in different shades of blue. **C.** Predicted binding poses of known compounds 2 to 4. In all cases the PM volume is shown but not the specific features to avoid crowding.

**Supplemental References**

1. Lee JY, Krieger J, Herguedas B, García-Nafría J, Dutta A, Shaikh SA, Greger IH, Bahar I (2019) Druggability Simulations and X-Ray Crystallography Reveal a Ligand-Binding Site in the GluA3 AMPA Receptor N-Terminal Domain. Structure 27:241-252.e243.

2. Bakan A, Nevins N, Lakdawala AS, Bahar I (2012) Druggability Assessment of Allosteric Proteins by Dynamics Simulations in the Presence of Probe Molecules. Journal of Chemical Theory and Computation 8:2435-2447.

3. Humphrey W, Dalke A, Schulten K (1996) VMD: visual molecular dynamics. J Mol Graph 14:33-38, 27-38.

4. Phillips JC, Braun R, Wang W, Gumbart J, Tajkhorshid E, Villa E, Chipot C, Skeel RD, Kale L, Schulten K (2005) Scalable molecular dynamics with NAMD. Journal of Computational Chemistry 26:1781-1802.

5. Sunseri J, Koes DR (2016) Pharmit: interactive exploration of chemical space. Nucleic Acids Research 44:W442-W448.
